# Magnetic Immobilization and Growth of *Nannochloropsis oceanica* and *Scenedasmus almeriensis*

**DOI:** 10.3390/plants11010072

**Published:** 2021-12-27

**Authors:** Maria G. Savvidou, Angelo Ferraro, Petros Schinas, Diomi Mamma, Dimitris Kekos, Evangelos Hristoforou, Fragiskos N. Kolisis

**Affiliations:** 1 Biotechnology Laboratory, School of Chemical Engineering, National Technical University of Athens, 9 Iroon Polytechniou Str, Zografou Campus, 15780 Athens, Greece; an.ferraro@libero.it (A.F.); dmamma@chemeng.ntua.gr (D.M.); kekos@chemeng.ntua.gr (D.K.); 2 Laboratory of Electronic Sensors, School of Electrical and Computer Engineering, National Technical University of Athens, 9 Iroon Polytechniou Str, Zografou Campus, 15780 Athens, Greece; hristoforou@ece.ntua.gr; 3 Environment and Quality of Life Center, School of Chemical Engineering, National Technical University of Athens, 9 Iroon Polytechniou Str, Zografou Campus, 15780 Athens, Greece; pschinas@mail.ntua.gr

**Keywords:** microalgae, *N. oceanica*, *S. almeriensis*, magnetic nanoparticles, immobilization, transformation, protoplast, cell growth

## Abstract

Microalgae are used in industrial and pharmaceutical applications. Their performance on biological applications may be improved by their immobilization. This study presents a way of cell immobilization using microalgae carrying magnetic properties. *Nannochloropsis oceanica* and *Scenedasmus almeriensis* cells were treated enzymatically (cellulase) and mechanically (glass beads), generating protoplasts as a means of incorporation of magnetic nanoparticles. Scanning electron microscopy images verified the successful cell wall destruction for both of the examined microalgae cells. Subsequently, protoplasts were transformed with magnetic nanoparticles by a continuous electroporation method and then cultured on a magnetic surface. Regeneration of transformed protoplasts was optimized using various organic carbon and amino acid supplements. Both protoplast preparation methods demonstrated similar efficiency. Casamino acids, as source of amino acids, were the most efficient compound for *N. oceanica* protoplasts regeneration in enzymatic and mechanical treatment, while for *S. almeriensis* protoplasts regeneration, fructose, as source of organic carbon, was the most effective. Protoplasts transformation efficiency values with magnetic nanoparticles after enzymatic or mechanical treatments for *N. oceanica* and *S. almeriensis* were 17.8% and 10.7%, and 18.6% and 15.7%, respectively. Finally, selected magnetic cells were immobilized and grown on a vertical magnetic surface exposed to light and without any supplement.

## 1. Introduction

Microalgae are photosynthetic microorganisms that are extensively used in pharmaceutical and industrial applications as a rich source of pigments, fatty acids, oils, anti-oxidants, food supplements, and lipids for biofuel production [1,2]. Restrictions regarding productivity and cost during isolation of the above-mentioned high-added value products headed towards new tools for biomass accumulation and enhanced metabolite production as well as towards more efficient isolation processes [3]. One of those new strategies is the protoplasts formation supporting the ability of microalgae cells to be genetically transformed, providing a foreign gene expression platform due to their quick growth upon photoautotrophic conditions [4,5,6]. Protoplasts also provide the advantage of studying protein–protein interactions and cell wall regeneration [7].

Protoplast, as a term, refers to a partial or total detached cell wall from bacteria, fungi, algae, and plant cells [8]. Microalgae cell walls differ from various microalgae species but are overall constituted by rigid components embedded in a polymeric matrix, which is composed of 70% cellulose as well as glycoproteins, pectin, and alganean, enhancing the persistence of cell walls [9].

*Nannochloropsis oceanica* (*N. oceanica*) is one of the six members of the *Nannochloropsis* microalgae family producing high levels of pigments such as astaxanthin and zeaxanthin as well as bio-lipids used for biofuel production [10,11,12]. Higher bio-lipid concentrations can be achieved upon metabolic targeting or through the suitable growth medium upon P, N, Fe, or Mg depletion [13]. *Scenedasmus almeriensis* (*S. almeriensis*) is a member of the *Scenedasmus* microalgae family, which is used frequently for biofuel production and wastewater management, as well as being a rich lutein source that is related to eye diseases and diet [14,15,16].

The alcohol-insoluble *N. oceanica* (strain CCMP 1779) cell wall material is constituted by glucose, mannose, and lower quantities of rhamnose, fucose, arabinose, xylose, and galactose as well as alganeans [17,18]. Bioinformatics analysis on the same strain verified two enriched cell wall enzymes, both belonging to the cellulose synthase family, guiding us to treat enzymatically the *N. oceanica* strain with cellulase regarding protoplast formation [18]. The *Nannochloropsis* cell wall is constituted by an outer alganean layer and an inner one that is constituted by 75% cellulose and also 6% of proteins [19]. The *Scenedasmus* cell wall is composed of cellulose and glucose in the inner layers and alganeans in the outer membrane as well as glycoproteins and pectin [20,21,22], with the previously referred constituents being 30% of the total wall compounds [23].

Enzymatic methods, such as algae cell treatment with cellulase, hemi-cellulase, and pectinase or mixtures of them, or mechanical methods, such as sonication and microwaves, as well as glass beads agitation, are the most frequently used techniques for protoplast formation [24,25,26]. Factors such as cell wall thickness, temperature, enzymes incubation time, pH, agitation, and the nature of the osmotic solution can affect protoplast formation [27]. Since protoplast formation is a stress-controlled process, re-creation of a fully structured and functional cell should be followed by treatment aiming at efficient regeneration of the cell walls [28]. Previous efforts based on lysozyme treatment on *Nannochloropsis limnetica*, polyethylene glycol treatment on *Nicotiana tabacum*, or enzyme mix on *Nannochloropsis* being used for microalgae protoplasts formation targeting cell wall removal *sp.* had encountered the field of protoplast generation [29,30,31].

The great potential of microalgae to be used as cell-factories directed us in this study to examine the efficacy of single enzymatic (cellulase) digestion as well as mechanical (glass bead bombardment) treatments on *N. oceanica* and *S. almeriensis* protoplast formation for further cell manipulation. The selection of these two specific microalgae strains was based on the fact that the *S. almeriensis* is not so well studied compared with other microalgae regarding protoplast generation, and furthermore, both of them are rich sources of high added-value products. Using scanning electron microscopy (SEM) visualization, as well as the cell wall regeneration based on different extra carbon and amino acids sources in short- and long-term action, we report how protoplasts can be used for biotechnological applications. In particular, we display how to introduce in their cytosol magnetic nanoparticles to confer magnetic properties to living *N. oceanica* and *S. almeriensis*. In the present work, we demonstrate that after nanoparticle introduction, microalgae cells are viable and can grow immobilized onto a magnetic surface.

## 2. Results and Discussion

### 2.1. Enzymatic and Mechanical Treatment of N. oceanica and S. almeriensis Microalgae Cells Driving Protoplasts Formation

**Figure 1:**
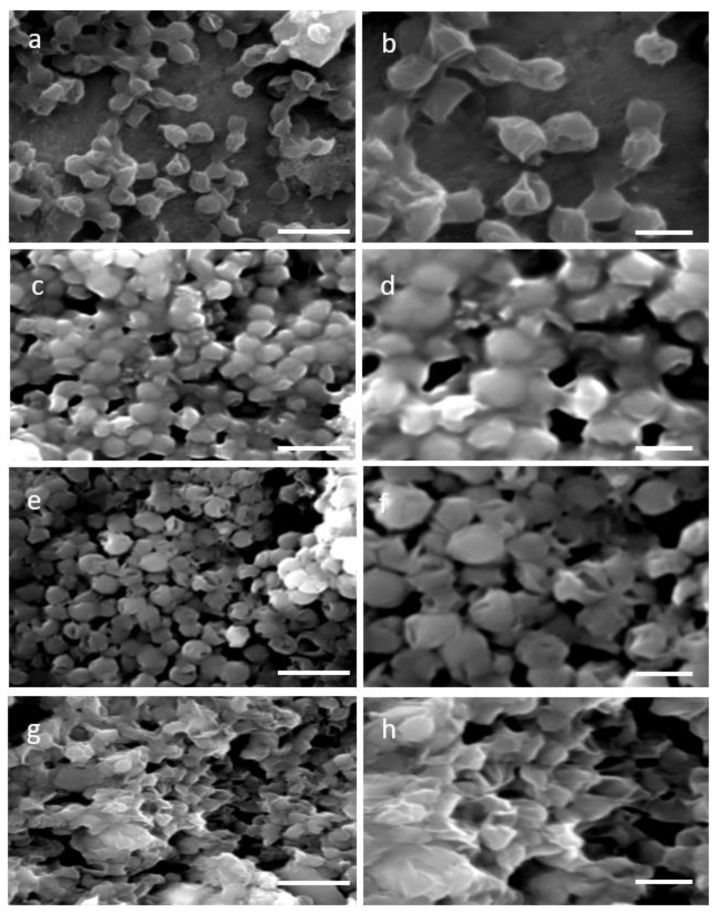
Scanning electron micrographs (SEM) for two different magnifications (5000× **left column**, 10,000× **right column**) of: (**a**,**b**) untreated cells (control) of *N. oceanica*; (**c**,**d**) digested cells of *N. oceanica* with cellulase for 4 h; (**e**,**f**) digested cells of *N. oceanica* with cellulase for 16 h; (**g**,**h**) mechanical pretreated cells of *N. oceanica* with glass beads. Scale bars: 5 µm left column, 2 µm right column.

**Figure 2:**
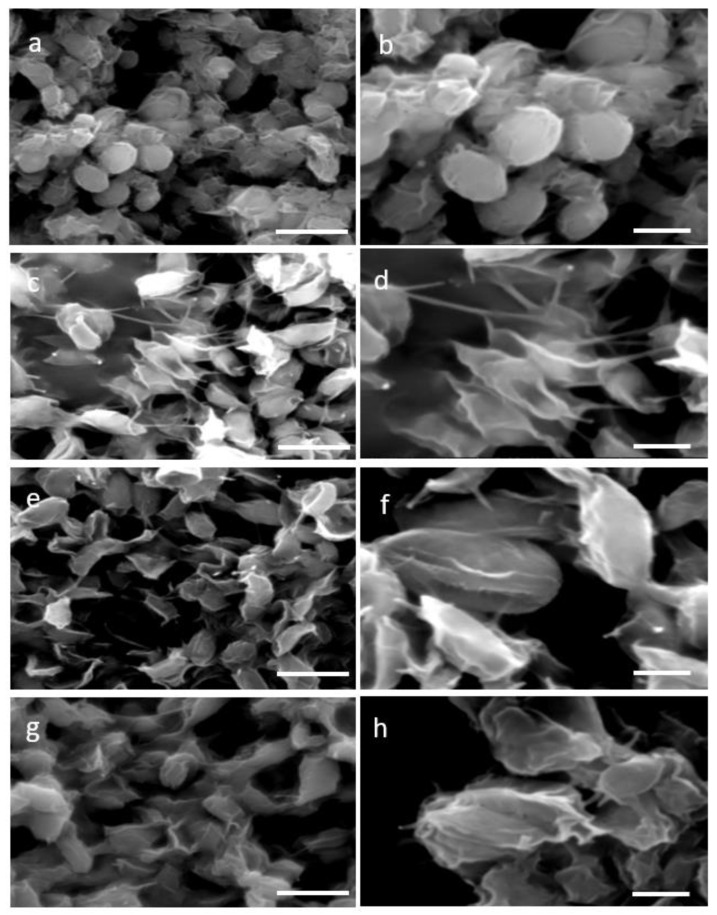
Scanning electron micrographs (SEM) for two different magnifications (5000× **left column**, 10,000× **right column**) of: (**a**,**b**) untreated cells (control) of *S. almeriensis*; (**c**,**d**) digested cells of *S. almeriensis* with cellulase for 4 h; (**e**,**f**) digested cells of *S. almeriensis* with cellulase for 16 h; (**g**,**h**) mechanical pretreated cells of *S. almeriensis* with glass beads. Scale bars: 5 µm left column, 2 µm right column.

Two distinctive methodologies for protoplast formation—the enzymatic, which is based on cellulase treatment and the mechanical, specifically the glass beads agitation process—were used aiming at cell wall removal. Four time points 4 h, 8 h, 12 h, and 16 h of single enzyme treatment resulted in a partial wall degradation and thus a more permeable and less rigid cell wall. Visualization of the stressed cell morphology due to the cell wall degradation was accomplished by SEM images of 4 h and 16 h of enzymatic as well as mechanical treatment. SEM images of *N. oceanica* untreated cells verified their complete structure (Figure 1a,b), while after the cellulase treatment the damaged cell wall was evident (Figure 1c–f) compared with the smoother surface and regular surface of the round untreated cells. The cell wall damage appearing at the two different incubation time periods had almost the same morphological changes. A more consistent cell wall destructive grade of *N. oceanica* cells was achieved by the glass beads mechanical treatment, as can be seen by SEM image (Figure 1g,h). Cells appear more stressed and smaller in size, most probably due to a more significant loss of cytoplasmic material during SEM sample preparation as the result of cell wall damage. The same cellulase treatment protocol used for *N. oceanica*, in *S. almeriensis* resulted in efficient cell wall degradation, as can be seen by SEM images in Figure 2c–f. Cells are less spherical and appeared more stretched in shape. The mechanical treatment of *S. almeriensis* resulted in similar morphological changes compared with the enzymatic process (Figure 2g,h). The success rate of protoplast preparation was average, and it did not differ significantly amongst the various treatments, as this can be verified by the SEM images, while mechanical treatment gave better results in comparison with the enzymatic treatment.

### 2.2. Protoplasts Regeneration Using Carbon and Amino Acids Supplements

The efficiency of cell wall reconstitution and regeneration after protoplast preparation was studied by adding extra carbon and amino acid sources into microalgae growth media. We reasoned that after cell wall removal, several pathways might not be properly supplied by solely photosynthetic activity, and thus might delay the cell wall reconstruction and in turn the full recovery of the cells. Particularly, supplementation with 1% *w/v* and 2% *w/v* glucose and 1% *w/v* and 2% *w/v* fructose, as well as 0.1% *w/v* casamino acids to the growth media separately, was examined. Recovery trends were calculated by measuring microalgae cell growth every two to three days, measuring the cell density (OD_750nm_ for *N. oceanica* and OD_600nm_ for *S. almeriensis*) up to 12 days in total. Due to different microalgae cell concentration in every experiment, the recovery trends were generated by standardizing spectrophotometer data via dividing every OD_750nm_ (or OD_600nm_) measurement with the initial OD_750nm_ (or OD_600nm_).

**Figure 3:**
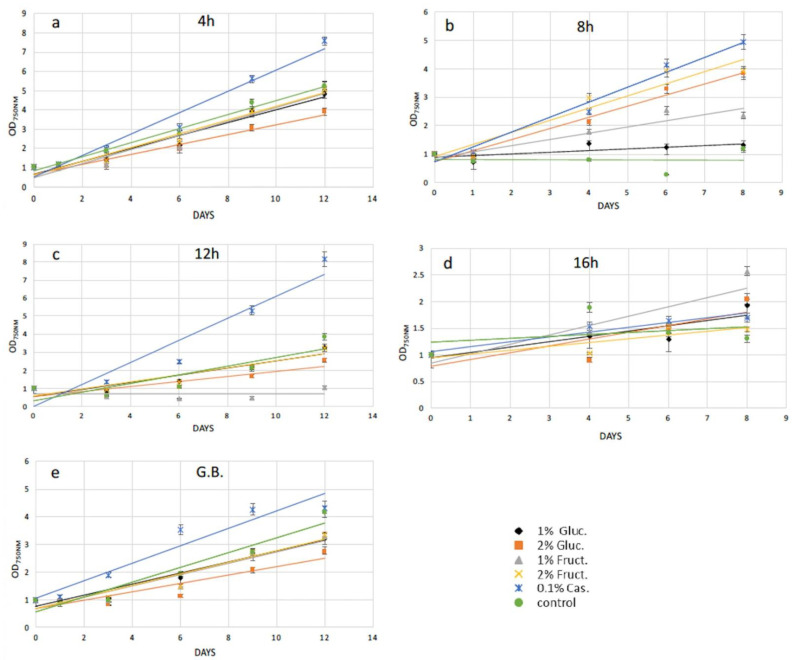
Recovery trends of pretreated (enzymatic and mechanical treatment) microalgae cells of *N. oceanica* in media with various organic carbon and amino acid supplements for (**a**) digested cells with cellulase for 4 h; (**b**) digested cells with cellulase for 8 h; (**c**) digested cells with cellulase for 12 h; (**d**) digested cells with cellulase for 16 h; (**e**) pretreated cells with glass beads (mechanical treatment). G.B., glass beads.

**Figure 4:**
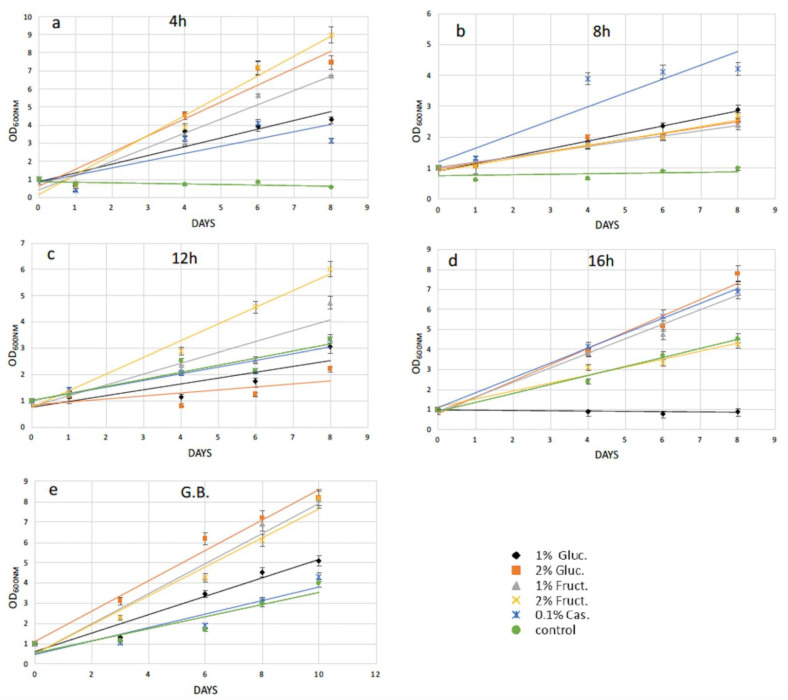
Recovery trends of pretreated (enzymatic and mechanical treatment) microalgae cells of *S. almeriensis* in media with various organic carbon and amino acid supplements for (**a**) digested cells with cellulase for 4 h; (**b**) digested cells with cellulase for 8 h; (**c**) digested cells with cellulase for 12 h; (**d**) digested cells with cellulase for 16 h; (**e**) pretreated cells with glass beads (mechanical treatment). G.B., glass beads.

Following 4 h of cellulase treatment for *N. oceanica*, casamino acids enhanced the recovery compared with the cells grown without any extra additions, while the rest of the added compounds could not support a better growth for the *N. oceanica* protoplasts (Figure 3a). A different trend was revealed in *S. almeriensis*, referring to the same enzymatic time period incubation, where 2% *w/v* fructose was the most successful compound to promptly restart cell growth after the wall digestion (Figure 4a). However, 2% *w/v* glucose and 1% *w/v* fructose for *S. almeriensis* were the second most successful group of compounds, considering cell wall regeneration followed by 1% *w/v* glucose and 0.1% *w/v* casamino acids (Figure 4a). After 8 h of *N. oceanica* enzymatic treatment, 0.1% *w/v* of casamino acids resulted in the best cell revitalization. A portion of 2% *w/v* of glucose and fructose enhanced the recovery trend efficiency of protoplasts compared with 1% *w/v* (Figure 3b). Casamino acids were still the leading compound for *S. almeriensis* protoplasts restoration, while all the rest of the compounds supported the regeneration of protoplasts in the same grade after 8 h of cellulase treatment (Figure 4b).

Casamino acids enhanced the *N. oceanica* protoplasts regeneration after 12 h, as for 4 h of enzymatic digestion, while all the rest showed a slightly reduced recovery compared with the cells grown in their absence (Figure 3c). Only 2% *w/v* fructose for *S. almeriensis* at the same cellulase treatment demonstrated the best revitalization trend after 8 days of growth compared with the cells grown without an extra carbon or amino acid source (Figure 4c).

Fructose at a concentration of 1% *w/v* was the most essential compound for *N. oceanica* protoplasts regeneration for 8 days of growth, after the longest time period of cellulase treatment of 16 h, while the rest of the compounds revealed the same efficacy, with 2% *w/v* fructose showing a slightly reduced recovery efficiency, compared with the rest of the group (Figure 3d). *S. almeriensis* demonstrated a different behavior at 16 h for enzymatic treatment compared with *N. oceanica*, since 2% *w/v* glucose, 1% *w/v* fructose, and 0.1% *w/v* casamino acids enhanced the recovery trend with the same success after 8 days of growth, while 2% *w/v* fructose did not provide any improvement compared with the cells grown without extra carbon or amino acid sources. Glucose at 1% *w/v* inhibited even more of the regeneration efficacy after 8 days of growth (Figure 4d).

Glass beads agitation stressed *N. oceanica* cells to such a degree that only 0.1% *w/v* casamino acids managed to enhance the recovery trend (Figure 3e); however, for *S. almeriensis*, the casamino acids did not enhance the recovery trend efficiency. Moreover, regarding the *S. almeriensis*, the concentration of 1% *w/v* glucose slightly increased regeneration, with 2% *w/v* glucose and fructose being the most effective compounds as well as 1% *w/v* fructose (Figure 4e). Overall, the glass beads agitation was more detrimental for *N. oceanica* because, regardless of used supplement, after 10 days of recovery the max recorded OD was ~4, whereas for *S. almeriensis*, the max OD after 10 days of recovery was ~8 (Figure 3e and Figure 4e).

### 2.3. Transformation of N. oceanica and S. almeriensis Protoplasts with Magnetic Nanoparticles and Their Cultivation

Successfully generated protoplasts possess the ability for an efficient transformation. For this reason, protoplasts from both microalgae species were transformed with magnetic nanoparticles through a continuous electroporation method described previously [32]. After cell wall removal, cells were immediately subjected to electroporation and were subsequently recovered in supplemented media based on results presented in the previous paragraph.

**Figure 5:**
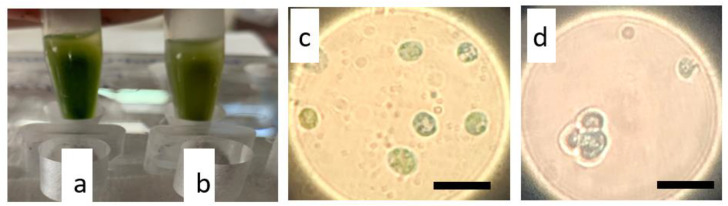
Separation of magnetic cells and visualization of internalized magnetic iron oxides nanoparticles by light microscopy after Prussian blue staining. (**a**) *N. oceanica* and (**b**) *S. almeriensis* magnetic cells, 3 days after electroporation, were selected using a magnetic rack that attracts magnetic cells at the tube walls; in (**c**) *N. oceanica* and (**d**) *S. almeriensis*, a magnification of magnetic cells stained in blue is reported; a digital zoom was used. Scale bar 10 µm.

Prussian blue staining verified the uptake of magnetic nanoparticles into *N. oceanica* and *S. almeriensis* cells. To perform this assay, small aliquots (1 mL) of cultures were used. Magnetic cells were isolated from the rest of the culture using a magnetic rack (Figure 5a,b) and stained with Prussian blue reagent that in the presence of iron turns blue. Blue-stained aggregates of iron oxide nanoparticles were observed within the cytoplasm of both microalgae cells that confirmed a successful nanoparticles internalization (Figure 5c,d). The transformation efficiency was calculated based on electroporation and recovery efficiency for both treatments and both examined microalgae strains. The measured transformation efficiency (at the third day after the electroporation and protoplasts recovery), resulted in 17.8% of magnetically active cells for 8 h of enzymatic digestion, while glass bead bombardment achieved a lower percentage of 10.7% for the *N. oceanica* protoplasts (Table 1). A similar outcome was observed for *S. almeriensis* protoplasts obtained by enzymatic treatment, being the more efficient method (18.6%) compared with glass beads (15.7%) (Table 2).

**Figure 6:**
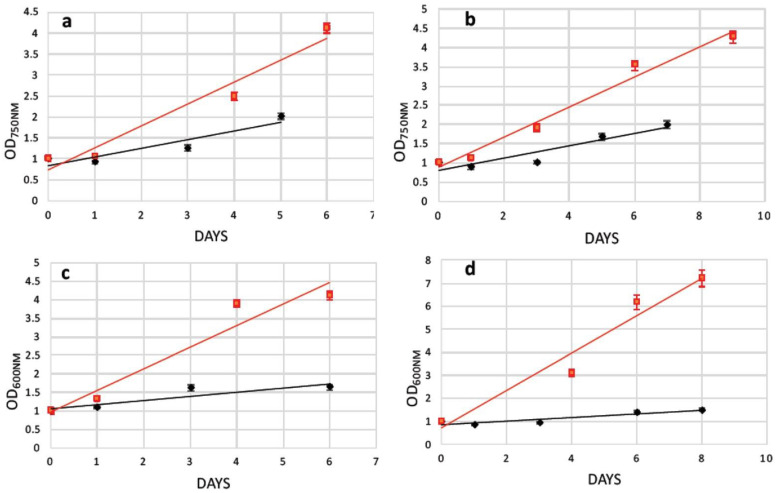
Recovery trends of pretreated microalgae cells (red lines) versus pretreated and electroporated microalgae cells (black lines). (**a**) Growth comparison of *N. oceanica* protoplasts pretreated with cellulase (8 h) in media supplemented with 0.1% *w/v* casamino acids; (**b**) growth comparison of *N. oceanica* protoplasts pretreated with glass beads in media supplemented with 0.1% *w/v* casamino acids; (**c**) growth comparison of *S. almeriensis* protoplasts pretreated with cellulase (8 h) in media supplemented with 0.1% *w/v* casamino acids; (**d**) growth comparison *of S. almeriensis* protoplasts pretreated with glass beads in media supplemented with 2% *w/v* glucose.

As mentioned earlier, after electroporation cells were cultured in specific media for a fast cell wall reconstruction. The recovery trend lines of the transformed *N. oceanica* and *S. almeriensis* protoplasts upon the assist of 0.1% *w/v* casamino acids (or 2% *w/v* glucose for *S. almeriensis* protoplasts’ pretreated with glass beads) were calculated in flask without magnetic surface and showed that the enzymatic-treated cells (Figure 6a–d black lines) could be regenerated slightly faster than those treated with glass beads, even in the absence of the additional stress provoked by electroporation (Figure 6 red lines). Subsequently, after 5 or 6 days cultivated in recovery media, magnetically responsive cells were separated by those that were poorly magnetic or those that did not uptake magnetic nanoparticles. This procedure was performed manually using a strong permanent magnet to select magnetic cells in sterile tubes; an example of magnetic separation can be seen in Figure 5a,b.

**Figure 7:**
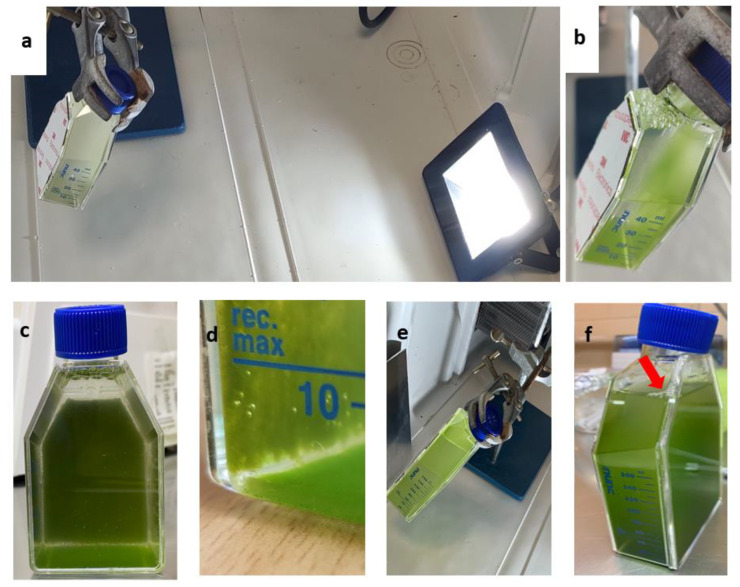
Vertical cultivation of *N. oceanica* (**a**–**d**) and *S. almeriensis* (**e**,**f**) magnetic cells immobilized on flasks with magnetic surface and exposed to LED light. (**a**) *N. oceanica* and (**e**) *S. almeriensis* at 1st day of cultivation; (**b**–**d**) *N. oceanica* at 3rd day of cultivation with molecular oxygen generation (gas bubbles in **d**); note that the majority of microalgae cells (magnetic) do not fall on the flask bottom; (**f**) *S. almeriensis* at 3rd day cultivation, the arrow indicates molecular oxygen generated during the growth.

Both treatments for protoplasts generation after the transformation provided living and metabolically active cells. A standard cell culture flask was modified by gluing a soft magnetic sheet (Figure 7a,b) on its back side. Selected magnetic cells were then added to the flask and left to attach on the magnetic side, leaving the flask in horizontal position for 10 min. As can be seen from Figure 7, cells remained attached to the magnetic surface on a flask tilted obliquely with respect to the light source. The light green color (Figure 7c–f), which coincides with the fully functional chlorophyll pigments of the microalgae layer generated on the magnetic surface, indicates fully viable and active cells. Furthermore, their viability can also be assessed by the gas (bubble) production that most probably is oxygen, even though we did not perform any analysis on the generated gas. Moreover, the transformed microalgae did not delay their recovery and magnetic growth compared with the two protocols, enzymatically or mechanically treated, to prepare protoplasts.

Finally, the growth of magnetically active cells from both *N. oceanica* and *S. almeriensis* was performed by measuring the optical density of the magnetic concentrated cells alone and after 3 days of cultivation. The optical density of the mixture of magnetic cells and the newly generated non-magnetic (daughter) cells resulted in an increase in growth of 24% and 16% for *N. oceanica* and *S. almeriensis* (Table 3).

## 3. Discussion

Microalgae cell wall structure and composition governs cell growth, harvesting, compound isolation, and other basic algae processes. The barrier between microalgae and the environment created by the cell wall protects the cells from external pressure fluctuations and regulates their interaction with flocculants and rules mass transfer [33]. Cell wall degradation results in genetically or biotechnologically transformed microalgae, with a gain of new-desired properties [7]. In this report, we examined the *N. oceanica* as well as *S. almeriensis* protoplasts generation based on single-enzymatic and mechanical approaches and the respective protoplast regeneration upon extra carbon or amino acid sources, as well as their potential transformation properties, introducing magnetic nanoparticles in the microalgae cell body. Additionally, the relevance of the microalgae manipulation was shown by demonstration that after nanoparticles introduction, microalgae cells were able to grow in an unconventional fashion.

Based on the rich cellulose compartments of the cell wall in both microalgae species, single-enzyme treatment (cellulase), which degrades cellulose as a linear polymer of D-glucose with β-1,4 linkages, and a mechanical approach were used for protoplasts generation. Different time periods of cellulase treatment, from 4 h to 16 h, demonstrated by SEM images analysis the same morphological protoplast formation efficiency in both microalgae species, with *Scenedasmus* revealing a better destructive cell wall compared with *Nannochloropsis*. However, the metabolic activity was slightly different between the two strains with respect to digestion time, as well as to recovery media. We optimized the most efficient protoplasts generation with single enzymatic incubation and in shorter time periods in both microalgae species, in order to provide the fastest and most economically favorable way of generating microalgae cells with a partially removed cell wall, compared with previous studies on *Dictyopteris prolifera*, where a mixture of cellulose with other enzymes and longer time periods of treatment delivered the optimal cell wall digestion [34]. The shorter enzymatic period of treatment in our study is in agreement with multi-enzymatic treatments in *Gracilaria* and *Ulva* species [35,36]. Physiological and biochemical changes between the cell walls of various microalgae species rule the protoplast formation yields [37]. Single-enzymatic treatments (pectinase) of *Scenedesmus quadricauda*, or combination of them with sonication and multiple-enzymatic treatments (hemicellulose and driselase minus/plus lysozyme, respectively) for *Nannochloropsis oculata* and *Nannochloropsis limnetica* performed high protoplast generation yields in a short time period treatment [29,38,39]. The temperature of 30 °C used in our studies throughout the cellulase treatment of *Nannochloropsis* and *Scenedasmus* microalgae uncovered optimal cell wall digestion efficacy, as in various *Chlorella* species [40]. The same temperature delivered the optimum cell wall digestion for *H. pluvialis* cells [32]. In *Gracilaria* and *Ulva* species for protoplast generation, a temperature range between 20 and 25 °C was used [36].

Proper osmotic conditions led to enhanced protoplast generation yields and viability, as well as regeneration, protecting the fragile cell from bursting or shrinking. Usually mannitol and sorbitol, as well as sodium chloride, increased protoplasts yields [36], supporting mitosis and daughter cell formation and protecting protoplasts from osmotic shocks [27]. Studies on *Chlorophyta* species revealed cell membrane regeneration 12 h after wounding, supported by an extensive protein expression governed by Golgi apparatus [41], and furthermore, cell-wall-digested cells of *Bryopsis plumose* cells developed in 9–12 h a lipid membrane demonstrating a regeneration efficiency of 40% [42]. In both microalgae strains examined in this study, mannitol was used together with cellulase and efficiently supported regeneration, preventing undesirable osmotic shock.

Glass beads agitation resulted in a higher efficiency in protoplasts formation compared with the enzymatic process for *Nannochloropsis* and *Scenedasmus* species after 30 s of vortex. Vortexing time is related to microalgae survival and regeneration after the mechanical treatment [4].

The next step following protoplast formation was to stimulate a fast and efficient regeneration of the microalgae cell-wall, leading to structure-complete mature cells. At the beginning of the research, we did not use any supplement after enzymatic or mechanical treatments, verifying that after a few days cells were not viable. Therefore, we hypothesized that the cell wall removal could affect their photosynthetic ability. For this reason, we used the simplest form of sugars and amino acids in order to help cells to fully regain photosynthetic ability and reconstruct the cell wall. The use of an excess amount of glucose or fructose may support a direct use of these compounds on the regenerated cell wall structure, as well as activation of glycolysis. Specifically, activation of glycolysis through fructose as its intermediate metabolite led to cellulose production by conversion of fructose-6-phosphate to glucose-6-phosphate via phosphoglucose isomerase, and in turn, cellulose can support a proper reconstruction of the microalgae cell wall. On the other hand, the activation of protein synthesis by casamino acids may support the expression of all the necessary proteins for a fully regenerated cell wall. Casamino acids and fructose were the crucial compounds for *N. oceanica* cell wall regeneration. Specifically, casamino acids showed the highest regeneration efficiency in 4 h, 8 h, and 12 h of cellulase treatment, followed by 1% *w/v* fructose for 16 h. Regarding *S. almeriensis*, 2% *w/v* fructose was the leading compound for protoplast regeneration at 4 h and 12 h of cellulase treatment, while casamino acids was the leading compound at 8 h. Glucose 2% *w/v* and fructose 1% *w/v*, as well as casamino acids, had the same protoplast regeneration efficiency at 16 h of enzymatic treatment. The two examined microalgae species demonstrated a high percentage of similarity regarding the compounds, leading to the most successful regeneration since fructose and casamino acids are the critical compounds for cell wall reconstruction. While casamino acids demonstrated an enhanced regeneration efficacy for the cells grown without any additional added compound in *N. oceanica*, 2% *w/v* glucose was the most essential compound for *S. almeriensis* protoplast regeneration upon glass beads agitation. Despite the highest protoplast formation efficacy of mechanical versus enzymatic treatment, casamino acids were the essential compound for both protoplast formation protocols for *N. oceanica*, except for 16 h of enzymatic treatment, while fructose as well as glucose were the leader compounds for protoplast regeneration regarding mechanical treatment as well as for all the different time periods of cellulase treatments for *S. almeriensis*, except for the 8 h incubation. Concluding, casamino acids as well as fructose were the most essential compounds for *N. oceanica* and *S. almeriensis* after short-term cellulase treatment or glass beads agitation processes.

Another important result of our work was the generation of magnetically active cells that could be used as means to immobilize cells for biocatalysis and biotechnological applications, such as a magnetic photobioreactor with continuous biomass production, having the advantage of using low water volumes. Cell immobilization, which represents the restriction of cell mobility by chemical or physical means is achieved by several strategies and allows the enzymatic and metabolic pathways of living cells to be fully exploited [43]. Up to now, various immobilization techniques had been used—either active or passive. The passive immobilization harnesses the natural capability of microorganisms of attaching to solid and gelatinous surfaces via chemical or physical mechanisms and is a less stressful technique than the active immobilization method, which is based on covalent binding or entrapment of cells on gels such as alginate. *Scenedasmus quadricauda* had been trapped on polyurethane foam cubes via the passive immobilization technique. In general chitin, chitosan, agarose, and agaropectin are used for cell entrapment [44]. Even though advanced, the majority of immobilization methods suffer from a similar drawback: the newly generated cells tend to remain in culture, resulting in system oversaturation. In our case, the magnetic immobilization of cells allowed this limitation to be overcome and at the same time kept all the advantages of such a technique. Indeed, in immobilized magnetic cells during cell division, only one of the daughter cells will retain the majority of magnetic nanoparticles because the magnetic gradient of the surface does not allow nanoparticles to be equally split (they are polarized toward the surface). Therefore, part of newly generated cells is not magnetic and therefore free to move in the growth medium. By gently removing the growth medium, the non-magnetic cells are removed as well, leaving a parental magnetic whole-cell population that can be used for biocatalytic production processes since the magnetic immobilized cells do not demonstrate any growth or viability defects [45]. Finally, it is important to mention that our work set the base for a new kind of cell immobilization method since magnetic nanoparticles could also be introduced in other kinds of microbial cells, such as bacteria, fungi, and other single cells microorganisms.

## 4. Materials and Methods

### 4.1. Strains and Growth Conditions

*Nannochloropsis oceanica* (CCMP1779) was obtained from the Provasoli-Guillard National Center for Culture of Marine Phytoplankton and cultured in sterilized seawater enriched with F/2 medium nutrients under aseptic conditions, as described elsewhere [13]. The cultivations were carried out in flasks at 20 °C with a continuous illumination at 100 μmol photon m^−2^ s^−1^.

*Scenedesmus almeriensis* was provided from Dr. Antonio Molino (ENEA, Italy) and cultured in modified Mann & Myers medium [46] with the following composition: 1.0 g/L NaNO_3_, 0.1 g/L K_2_HPO_4_, 1.2 g/L MgSO_4_*7H_2_O, and 0.3 g/L CaCl_2_. Additionally, 10 mL of a micronutrients solution, consisting of 0.001 mg/L Na_2_EDTA, 1.4 mg/L MnCl_2_, 0.33 mg/L ZnSO_4_*7H_2_O, 2 mg/L FeSO_4_*2H_2_O, 0.002 mg/L CuSO_4_*5H_2_O, and 0.007 mg/L Co(NO_3_)_2_*6H_2_O, was added to 990 mL of the medium. *S. almeriensis* cultures were grown in flasks at 26 °C with a continuous illumination at 60–80 μmol photon m^−2^ s^−1^. All experiments were conducted in triplicate.

### 4.2. Protoplast Preparation

#### 4.2.1. Enzymatic Cell Wall Digestion

The enzymatic cell wall digestion was performed as previously described [32]. In summary, *N. oceanica* and *S. almeriensis* cells (3 × 10^9^ cells/mL) were collected from log-phase cultures, after 6–8 days of cultivation, via centrifugation at 1000 *g* for 10 min. The cells were re-suspended in 30 mL of 50 mM phosphate buffer (pH. 7.0) containing 0.6 M D-mannitol and the enzyme cellulase (2% *w/v*) and were incubated for either 4, 8, 12, and 16 h at 30 °C. The solution for each strain was centrifuged at 900× *g* for 10 min. The pellet was re-suspended in the sugar solution (0.6 M D-mannitol), washed twice, and re-suspended again in 20 mL of medium containing no detergents, avoiding any protoplast rupture. Each treatment was carried out in triplicate.

#### 4.2.2. Glass Beads Cell Wall Disruption

The cell digestion was performed as previously described [32]. In summary, log-phase cells from microalgae *N. oceanica* and *S. almeriensis* were harvested, washed three times, and re-suspended in 50 mM phosphate buffer (pH 7.0) containing 0.6 M D-mannitol with no detergents, avoiding any protoplast rupture. Of these cells, 1 mL cells (3 × 10^9^ cells/mL) was transferred to 2 mL Eppendorf tubes that contained a 200 mg dry acid-washed glass beads of 1.0 mm (Sigma-Aldrich, St Louis, MO, USA). The cells were then disrupted by 30 s vigorous vortexing at 1500 rpm, and the supernatant of each sample was used for further analysis. Regarding blank controls, they were transformed without agitation. For all treatments, three independent agitations were performed.

### 4.3. Scanning Electron Microscopy Imaging for Observation of Morphological Changes in Cells

For morphological observation after the treatments for *N. oceanica* and *S. almeriensis* cell disruption, an FEI Quanta-200 scanning electron microscope (SEM) was applied for both enzymatic lyses and mechanical treatment. A similar fixation protocol and set up for the scanning electron microscope, as previously described [32], were used.

### 4.4. Nanoparticles

The superparamagnetic nanoparticles used in this study were fluidMAG-lipid magnetic nanoparticles coated in a surfactant, phosphatidylcholine, in order to be biocompatible and were supplied by Chemicell, GmbH (Berlin, Germany). The nanoparticles were autoclaved by the suppliers to ensure sterility. The material was used as received.

### 4.5. Transformation

The protoplasts of both microalgae *N. oceanica* and *S. almeriensis* were shocked at different field strengths (0.5–3.0 kV) before the electroporation to secure that the electroporation had not or had a low impact on the protoplast generation. For the transformation process, we followed the previously described protocol [32]. In summary, *N. oceanica* and *S. almeriensis* protoplasts were mixed with 100 μL fluidMAG-lipid magnetic nanoparticles, using medium containing 0.6 M D-mannitol solution as the electroporation buffer, and incubated for 30 min before the pulse. The transformation was performed by a homemade continuous flow cuvette device managing electroporation of high volumes, using a pulse generator -MicroPulser- from Bio-Rad (Hercules, CA, USA) and 1 pulse at 3 kV every 15 s and a peristaltic pump at a rate of ~100 µL per seconds. This flow-cuvette was constructed by 3D printing a plastic core on which aluminum plaques were glued, resulting in an internal volume of ~1.5 mL.

### 4.6. Protoplasts Regeneration before and after Electroporation

The ability of the *N. oceanica* and *S. almeriensis* protoplasts to regenerate was examined in F/2 and modified Mann & Myers medium, respectively. In summary, immediately after electroporation, the protoplasts of both strains were cultured at 20 °C and 26 °C, without agitation and at low light intensity ~60–70 μmol photon m^−2^ s^−1^ for up to twelve days in 5 mL growth medium supplemented with mannitol for osmotic stabilization of protoplasts, and different concentrations of carbon sources, glucose (1 and 2% *w/v*), or fructose (1 and 2% *w/v*) and nitrogen source such as casamino acids (0.01% *w/v*) to enhance the cell wall regeneration. The regenerated protoplasts were observed every 3 days by measuring the optical density at 750 nm and 600 nm, respectively, and with a light microscope at 40× magnification.

### 4.7. Transformation Efficiency

To compare the transformation efficiency of the *N. oceanica* and *S. almeriensis* after both treatments (enzymatic and mechanical) as well as the reconstruction of the cell walls, cells grew in the specific recovery medium for 3 days and then 1 mL for each condition was transferred to a 1.5 mL Eppendorf. The optical density OD_750nm_ and OD_600nm_ values for *N. oceanica* and *S. almeriensis*, respectively, were measured. Then, the same 1 mL sample was settled to the magnetic rack for 15 min in order for the magnetic cells to be bind on the magnetic part. Afterwards, the absorbance of the supernatant only for each strain was measured, and the value was lower compared with the initial value due to the magnetic cells that were previously attracted. The protoplast and electroporation efficiency was calculated by the rate between the two measurements.

### 4.8. Prussian Blue Staining

Prussian blue staining was carried out via the Biopal protocol (BioPhysics Assay Laboratory, Worcester, MA, USA) on fixed (methanol/acetone solution (7:1) for 10 min at room temperature) *N. oceanica* and *S. almeriensis* cells. Images with stained cells for both strains were obtained using a microscope.

### 4.9. Magnetic Cells Cultivation

In order to examine the generation of magnetically active microalgae, a standard cell culture flask was modified by gluing a soft magnetic sheet on its back side. Via a spiral, a permanent magnet, and a peristaltic pump, the magnetic cells were separated from non-magnetic cells and were then added to the flask and were left to attach on the magnetic side, leaving the flask in horizontal position for 10 min. The flasks were incubated at 20 °C and 26 °C at low light intensity ~60–70 μmol photon m^−2^ s^−1^ for 3–4 days. On day 3, the magnetic cells and the produced non-magnetic cells were mixed, and the optical density was measured at 750 nm and 600 nm, respectively, and compared with the initial optical density of only magnetic cells.

## 5. Conclusions

This study presented protoplast generation protocols using enzymatic (cellulase) or mechanical (glass beads agitation) treatments of *N. oceanica* and *S. almeriensis* microalgae cells, followed by the identification of the most efficacious compounds for the regeneration of microalgae cell walls. Regardless of various other studies, this work is the first that describes the efficiency of protoplasts generation in the two used microalgae strains, while attempting to examine the most efficient regeneration method of the produced protoplasts. Moreover, most of the studies till now used mixtures of enzymes without comparing results with other alternatives. We compared the enzymatic with the mechanical treatment, and we managed to reach protoplast generation efficiency via a single enzymatic treatment, similar to other work that used enzyme mixtures. Protoplast transformation efficiency values with magnetic nanoparticles after enzymatic or mechanical treatments for *N. oceanica* and *S. almeriensis* were 17.8% and 10.7%, and 18.6% and 15.7%, respectively, which can be improved by using better electroporation devices. The magnetic properties supported the successful immobilization and growth of microalgae cells on a vertical magnetic surface exposed to light and without any supplement. The novelty of our study was also focused on the immobilization method since the majority of these methods suffer from a similar drawback: newly generated cells tend to remain in culture, resulting in the system’s oversaturation. In our case, the magnetic immobilization of cells allowed us to overcome this limitation (new cells fell to the bottom of the flask and could be easily removed) and at the same time kept all the advantages of such technique. Furthermore, the immobilization of cells on polymers could be more stressful than on magnets, so our method in terms of generation of magnetically immobilized cells demonstrates an advantage compared with other immobilization techniques.

## Figures and Tables

**Table 1:** Transformation efficiency of *N. oceanica* protoplasts.

Conditions	OD_750nm_ (3rd Day after Recovery)	OD_750nm_ (After Magnetic Separation)	Transformation Efficiency (%)
**Enzymatic digestion (8 h)**	0.610	0.501	17.8
**Mechanical treatment**	0.912	0.825	10.7

Values are the means of three measurements, and the standard deviation was below 5% in all cases.

**Table 2:** Transformation efficiency of *S. almeriensis* protoplasts.

Conditions	OD_600nm_ (3rd Day after Recovery)	OD_600nm_ (After Magnetic Separation)	Transformation Efficiency (%)
**Enzymatic digestion (8 h)**	0.834	0.679	18.6
**Mechanical treatment**	0.670	0.565	15.7

Values are the means of three measurements, and the standard deviation was below 5% in all cases.

**Table 3:** Growth for vertical cultivation of *N. oceanica* and *S. almeriensis* magnetic cells.

Strains	Optical Density (1st Day)	Optical Density (3rd day)	Increment
***N. oceanica* (OD_750nm_)**	1.001	1.314	+24%
***S. almeriensis* (OD_600nm_)**	1.097	1.305	+16%

Values are the means of three measurements and the standard deviation was below 5% in all cases.

## Data Availability

Data are available under request.

## References

[1] Harun R., Singh M., Forde G.M., Danquah M.K. (2010). Bioprocess engineering of microalgae to produce a variety of consumer products. Renew. Sustain. Energy Rev..

[2] Koller M., Muhr A., Braunegg G. (2014). Microalgae as versatile cellular factories for valued products. Algal Res..

[3] Giraldo-Calderón N.D., Romo-Buchelly R.J., Arbeláez-Pérez A.A., Echeverri-Hincapié D., Atehortúa-Garcés L. (2018). Microalgae biorefineries: Applications and emerging technologies. DYNA.

[4] Coll J.M. (2000). Review. Methodologies for transferring DNA into eukaryotic microalgae. Span. J. Agric. Res..

[5] Doron L., Segal N., Shapira M. (2016). Transgene expression in microalgae—From tools to applications. Front. Plant Sci..

[6] Qin S., Lin H., Jiang P. (2012). Advances in genetic engineering of marine algae. Biotechnol. Adv..

[7] Faraco M., Di Sansebastiano G., Pietro S.K., Koes R.E., Quattrocchio F.M. (2011). One Protoplast Is Not the Other!. Plant Physiol..

[8] Verma N., Bansal M., Kumar V. (2008). Protoplast fusion technology and its biotechnological applications. Chem. Eng. Trans.

[9] Gerken H.G., Donohoe B., Knoshaug E.P. (2013). Enzymatic cell wall degradation of Chlorella vulgaris and other microalgae for biofuels production. Planta.

[10] Gouveia L., Oliveira A.C. (2009). Microalgae as a raw material for biofuels production. J. Ind. Microbiol. Biotechnol..

[11] Hibberd D.J. (1981). Notes on the taxonomy and nomenclature of the algal classes Eustigmatophyceae and Tribophyceae (synonym Xanthophyceae). Bot. J. Linn. Soc..

[12] Lubián L.M., Montero O., Moreno-Garrido I., Huertas I.E., Sobrino C., González-Del Valle M., Parés G. (2000). Nannochloropsis (Eustigmatophyceae) as source of commercially valuable pigments. J. Appl. Phycol..

[13] Savvidou M.G., Boli E., Logothetis D., Lymperopoulou T., Ferraro A., Louli V., Mamma D., Kekos D., Magoulas K., Kolisis F.N. (2020). A study on the effect of macro-and micro-nutrients on nannochloropsis oceanica growth, fatty acid composition and magnetic harvesting efficiency. Plants.

[14] Chan M.C., Ho S.H., Lee D.J., Chen C.Y., Huang C.C., Chang J.S. (2013). Characterization, extraction and purification of lutein produced by an indigenous microalga Scenedesmus obliquus CNW-N. Biochem. Eng. J..

[15] Mandal S., Mallick N. (2012). Biodiesel production by the green Microalga scenedesmus obliquus in a recirculatory aquaculture system. Appl. Environ. Microbiol..

[16] Mehariya S., Iovine A., Di Sanzo G., Larocca V., Martino M., Leone G.P., Casella P., Karatza D., Marino T., Musmarra D. (2019). Supercritical fluid extraction of lutein from *Scenedesmus almeriensis*. Molecules.

[17] Jeffree C.E. (2007). The Fine Structure of the Plant Cuticle. Annu. Plant Rev..

[18] Vieler A., Wu G., Tsai C.H., Bullard B., Cornish A.J., Harvey C., Reca I.B., Thornburg C., Achawanantakun R., Buehl C.J. (2012). Genome, Functional Gene Annotation, and Nuclear Transformation of the Heterokont Oleaginous Alga *Nannochloropsis oceanica* CCMP1779. PLoS Genet..

[19] Halim R., Hill D.R.A., Hanssen E., Webley P.A., Blackburn S., Grossman A.R., Posten C., Martin G.J.O. (2019). Towards sustainable microalgal biomass processing: Anaerobic induction of autolytic cell-wall self-ingestion in lipid-rich: Nannochloropsis slurries. Green Chem..

[20] Bisalputra T. (1965). The origin of the pectic layer of the cell wall of Scenedesmus Quadricauda. Can. J. Bot..

[21] Nemcova Y. (2003). Detection of cell wall structural polysaccharides by cellulase-gold complexes and Detekce polysacharidu v bunecne stene. Czecg Phycol..

[22] Voigt J., Stolarczyk A., Zych M., Malec P., Burczyk J. (2014). The cell-wall glycoproteins of the green alga Scenedesmus obliquus. The predominant cell-wall polypeptide of Scenedesmus obliquus is related to the cell-wall glycoprotein gp3 of Chlamydomonas reinhardtii. Plant Sci..

[23] Zhang Y., Kong X., Wang Z., Sun Y., Zhu S., Li L., Lv P. (2018). Optimization of enzymatic hydrolysis for effective lipid extraction from microalgae *Scenedesmus* sp.. Renew. Energy.

[24] Echeverri D., Romo J., Giraldo N., Atehortúa L., Echeverri D., Romo J., Giraldo N., Atehortúa L. (2019). Microalgae protoplasts isolation and fusion for biotechnology research. Rev. Colomb. Biotecnol..

[25] Ortiz-Matamoros M.F., Villanueva M.A., Islas-Flores T. (2018). Genetic transformation of cell-walled plant and algae cells: Delivering DNA through the cell wall. Brief. Funct. Genom..

[26] Spiden E.M., Scales P.J., Yap B.H.J., Kentish S.E., Hill D.R.A., Martin G.J.O. (2015). The effects of acidic and thermal pretreatment on the mechanical rupture of two industrially relevant microalgae: *Chlorella* sp. and *Navicula* sp.. Algal Res..

[27] Davey M.R., Anthony P., Power J.B., Lowe K.C. (2005). Plant protoplasts: Status and biotechnological perspectives. Biotechnol. Adv..

[28] Papadakis A.K., Roubelakis-Angelakis K.A. (2002). Oxidative stress could be responsible for the recalcitrance of plant protoplasts. Plant Physiol. Biochem..

[29] Noda J., Mühlroth A., Bučinská L., Dean J., Bones A.M., Sobotka R. (2017). Tools for biotechnological studies of the freshwater alga Nannochloropsis limnetica: Antibiotic resistance and protoplast production. J. Appl. Phycol..

[30] Gan Q., Jiang J., Han X., Wang S., Lu Y. (2018). Engineering the chloroplast genome of oleaginous marine microalga *Nannochloropsis oceanica*. Front. Plant Sci..

[31] Abidin A.A.Z., Suntarajh M., Yusof Z.N.B. (2020). Transformation of a Malaysian species of *Nannochloropsis: Gateway* to construction of transgenic microalgae as vaccine delivery system to aquatic organisms. Bioengineered.

[32] Savvidou M.G., Ferraro A., Hristoforou E., Mamma D., Kekos D., Kolisis F.N. (2020). Incorporation of Magnetic Nanoparticles into Protoplasts of Microalgae *Haematococcus pluvialis*: A Tool for Biotechnological Applications. Molecules.

[33] Scholz M.J., Weiss T.L., Jinkerson R.E., Jing J., Roth R., Goodenough U., Posewitz M.C., Gerken H.G. (2014). Ultrastructure and composition of the Nannochloropsis gaditana cell wall. Eukaryot. Cell.

[34] Fujimura T., Kawai T., Kajiwara T., Ishida Y. (1995). Protoplast isolation in the marine brown alga Dictyopteris prolifera (Dictyotales). Plant Cell Rep..

[35] Gupta V., Kumar M., Kumari P., Reddy C.R.K., Jha B. (2011). Optimization of protoplast yields from the red algae Gracilaria dura (C. Agardh) J. Agardh and G. verrucosa (Huds.) Papenfuss. J. Appl. Phycol..

[36] Reddy C.R.K., Gupta M.K., Mantri V.A., Jha B. (2008). Seaweed protoplasts: Status, biotechnological perspectives and needs. J. Appl. Phycol..

[37] Chen Y.C., Shih H.C. (2000). Development of protoplasts of Ulva fasciata (Chlorophyta) for algal seed stock. J. Phycol..

[38] Reshma R., Arumugam M. (2017). Selective degradation of the recalcitrant cell wall of Scenedesmus quadricauda CASA CC202. Planta.

[39] Ma X., Pan K., Zhang L., Zhu B., Yang G., Zhang X. (2016). Genetic transformation of Nannochloropsis oculata with a bacterial phleomycin resistance gene as dominant selective marker. J. Ocean Univ. China.

[40] Lu Y., Kong R., Hu L. (2012). Preparation of protoplasts from Chlorella protothecoides. World J. Microbiol. Biotechnol..

[41] Klochkova T.A., Kwak M.S., Kim G.H. (2016). Proteomic profiles and ultrastructure of regenerating protoplast of bryopsis plumosa (Chlorophyta). Algae.

[42] Kim G.H., Klotchkova T.A., Kang Y.M. (2001). Life without a cell membrane: Regeneration of protoplasts from disintegrated cells of the marine green alga Bryopsis plumosa. J. Cell Sci..

[43] Polakovič M., Švitel J., Bučko M., Filip J., Neděla V., Ansorge-Schumacher M.B., Gemeiner P. (2017). Progress in biocatalysis with immobilized viable whole cells: Systems development, reaction engineering and applications. Biotechnol. Lett..

[44] Vasilieva S.G., Lobakova E.S., Lukyanov A.A., Solovchenko A.E. (2016). Immobilized microalgae in biotechnology. Ecology.

[45] Tagizadeh S.M., Ebrahiminezhad A., Ghoshoon M.B., Dehshahri A., Berenjian A., Ghasemi Y. (2021). Impacts of magnetic immobilization on the growth and metabolic status of recombinant *Pichia pastoris*. Mol. Biotechnol..

[46] Mann J.E., Myers J. (1968). On pigments, growth, and photosynthesis of phaeodactylum tricornutum 2. J. Phycol..

